# Comparison of different methods to assess tacrolimus concentration intra-patient variability as potential marker of medication non-adherence

**DOI:** 10.3389/fphar.2022.973564

**Published:** 2022-10-13

**Authors:** Barbora Kostalova, Katerina Mala-Ladova, Sylvie Dusilova Sulkova, Kris Denhaerynck, Sabina De Geest, Josef Maly

**Affiliations:** ^1^ Department of Social and Clinical Pharmacy, Faculty of Pharmacy in Hradec Kralove, Charles University, Hradec Kralove, Czechia; ^2^ Department of Nephrology, University Hospital Hradec Kralove and Faculty of Medicine Hradec Kralove, Charles University, Hradec Kralove, Czechia; ^3^ Department Public Health, Institute of Nursing Science, University of Basel, Basel, Switzerland; ^4^ Academic Center for Nursing and Midwifery, Department of Public Health and Primary Care, KU Leuven, Leuven, Belgium

**Keywords:** immunosuppression, kidney transplantation, intra-patient variability, medication adherence, tacrolimus immunosuppression, tacrolimus

## Abstract

**Background and objective:** Non-adherence to tacrolimus commonly manifests as low drug concentrations and/or high intra-patient variability (IPV) of concentrations across multiple measurements. We aimed to compare several methods of tacrolimus IPV calculation and evaluate how well each reflects blood concentration variation due to medication non-adherence in kidney transplant recipients.

**Methods:** This Czech single-center retrospective longitudinal study was conducted in 2019. All outpatients ≥18 years of age, ≥3 months post-transplant, and on tacrolimus-based regimens were approached. After collecting seven consecutive tacrolimus concentrations we asked participating patients to self-report adherence to immunosuppressants (BAASIS^©^ scale). The IPV of tacrolimus was calculated as the medication level variability index (MLVI), the coefficient of variation (CV), the time-weighted CV, and *via* nonlinearly modeled dose-corrected trough levels. These patient-level variables were analyzed using regression analysis. Detected nonlinearities in the dose-response curve were controlled for by adding tacrolimus dosing and its higher-order terms as covariates, along with self-reported medication adherence levels.

**Results:** Of 243 patients using tacrolimus, 42% (*n* = 102) reported medication non-adherence. Non-adherence was associated with higher CVs, higher time-weighted CVs, and lower dose-corrected nonlinearly modeled trough levels; however, it was not associated with MLVIs. All of the significant operationalizations suggested a weak association that was similar across the applied methods.

**Discussion and conclusion:** Implementation non-adherence was reflected by higher CV or time-weighted CV and by lower blood concentrations of tacrolimus. As an additional tool for identifying patients at risk for non-adherence, simple IPV calculations incorporated into medical records should be considered in everyday clinical practice.

## 1 Introduction

Patients who undergo kidney transplantation (KTx) require lifelong immunosuppression. Maintenance immunosuppression includes a combination of medications, with tacrolimus-based regimens a top choice. Due to tacrolimus’ narrow therapeutic range and high pharmacokinetic variability, regular assessment of its concentration in the patient’s blood is necessary to guide tacrolimus management (Kidney Disease: Improving Global Outcomes Transplant Work Group, 2009). Its concentration varies both inter-individually [mainly due to demographic factors and metabolism on CYP450 (Gonzales et al., 2020)] and intra-individually [mainly due to medication non-adherence (Schumacher et al., 2021)].

Even small deviations in post-transplant medication adherence (>5%), i.e., the degree to which patients take their medication as prescribed, have been associated with an elevated risk of graft rejection (Butler et al., 2004; Gustavsen et al., 2019). Adherence consists of three phases: initiation, implementation, and discontinuation–each of which must be specifically assessed. Persistence is the length of time between initiation and the last dose, which immediately precedes discontinuation (Vrijens et al., 2012; Eliasson et al., 2020).

Immunosuppression is initiated before and during hospitalization. During this phase, as every dose is administered or supervised by a health care professional, non-adherence is not possible. It is in the following phase, implementation, that adherence becomes a critical issue, as this is when patients begin to establish the behaviors they will need for long-term self-management and persistence on the treatment. Approximately one-third of KTx patients begin to show non-adherence during their implementation phase. This proportion increases over time (De Geest et al., 2014). Treatment discontinuation is rare and can be assessed by drug monitoring if patients stay in follow-up (Neuberger et al., 2017).

During implementation, non-adherence to tacrolimus can manifest itself as low blood concentrations or as high intra-patient variability of concentrations (IPV) over several measurements (Rozen-Zvi et al., 2017). Simple IPV calculations, such as the medication level variability index (MLVI) or the coefficient of variation (CV), are commonly used in research (Schumacher et al., 2021). To separate dosing adjustments or timing influences on blood tacrolimus levels, both dose-adjusted (Kim et al., 2019) and time-adjusted (Rozen-Zvi et al., 2017) methods were proposed.

This study’s aim was to compare various methods of tacrolimus IPV calculations and evaluate how well each reflected blood tacrolimus concentration variation due to non-adherence to immunosuppressants in KTx recipients.

## 2 Materials and methods

### 2.1 Study design and setting

This single-center retrospective observational study was conducted in the outpatient transplant clinic of the University Hospital Hradec Kralove in the Czech Republic from May to December 2019. It was approved by the Ethics Committee of the University Hospital Hradec Kralove, and was conducted in accordance with the Helsinki and Istanbul Declarations.

The Czech healthcare system is a social health insurance system: all patients have free access to medical care. The Coordination Center for Transplantation allocates organs, manages transplant registries, and gathers regular statistics^1^. Seven transplant centers provide over 500 kidney grafts annually for the Czech Republic’s approximately 10 million inhabitants. The Transplantation Center in Hradec Kralove, where this study was conducted, performs approximately 50 KTx per year.

The frequency of follow-ups at the outpatient clinic varies mainly based on time since transplantation and each patient’s health status. Visits are scheduled several times for each of the first three months post-transplantation, then once per month for the rest of the year. In the second year post-transplant, the frequency varies from once each month to once every second month. From the beginning of the third year, the usual follow-up frequency is four times per year.

The first-choice maintenance immunosuppressive regimen is a combination of tacrolimus, mycophenolate mofetil, and corticosteroids. While medication costs are normally subject to limited surcharges, immunosuppressants (except corticosteroids) are fully covered.

### 2.2 Data collection

Data were collected by reviewing medical records and patient questionnaires. Participating patients were approached by a nephrology nurse during their scheduled visits.

### 2.3 Sampling methods

We first screened all consecutive patients at the outpatient transplant clinic for eligibility. All who were eligible were then approached for participation in the study. Inclusion criteria were ≥18 years of age, stable clinical status, ≥3months post-transplant, a tacrolimus-based immunosuppression regimen and provision of written informed consent. Patients not fluent in the Czech language, those suffering from severe cognitive or health impairment, as well as those on acute anti-rejective therapy or hospitalized were excluded. Re-transplantation was not an exclusion criterion.

### 2.4 Variables and measurement

#### 2.4.1 Socio-demographic and transplant variables

We assessed education level and working status through a structured written questionnaire. Age, gender and transplant characteristics, e.g., time post-transplant in years, donor type, type of KTx, current immunosuppressants, were all collected from medical records (detailed information in Table 1).

**TABLE 1:** Patient characteristics (*N* = 243).

Characteristic		Number
Patient characteristics		
Male (n,%)		165 (67.90%)
Age (median, IQR)	(in years)	56.75 (47.38–65.44)
Education (n,%)	Elementary	25 (10.29%)
	Secondary	184 (75.72%)
	Higher/professional school	8 (3.29%)
	University	25 (10.29%)
	Missing	1 (0.41%)
Working status* (n,%)	Working	112 (46.50%)
	Retired	73 (30.04%)
	Invalid	109 (44.86%)
Transplant characteristics		
Number of Tx (n (%))	First	212 (87.24%)
	Second	30 (12.35%)
	Third	1 (0.41%)
Time post-transplant (median, IQR)	(in years)	5.64 (2.79–10.30)
Donor type (n,%)	Cadaveric	222 (91.36%)
	Living unrelated	5 (2.06%)
	Living related	16 (6.58%)
Pre-emptive Tx (n,%)		29 (11.93%)
Immediate onset of kidney function (n,%)		187 (76.95%)
Rejection post-Tx (n,%)	<1-month post-Tx	29 (11.93%)
	≥1-month post-Tx**	48 (19.75%)
Tacrolimus-based immunosuppressive regimen (at the time of data collection)*		
+ Antiproliferative agents (n,%)	Mycophenolate mofetil or mycophenolic acid	222 (91.36%)
	Azathioprine	2 (0.82%)
+ mTOR inhibitors (n,%)	Sirolimus	5 (2.06%)
+ Corticosteroids (n,%)	Prednisone	225 (92.59%)
	Methylprednisolone	3 (1.23%)

* multiple answers possible.

** related to current transplantation, rejection leading to re-transplantation was not counted. IQR, interquartile range; N, denominator; Tx, transplantation.

#### 2.4.2 Self-reported medication adherence

Adherence to immunosuppressants (implementation phase) was assessed by the written version of the Basel Assessment of Adherence to Immunosuppressive Medications Scale (BAASIS^©^) (Dobbels et al., 2010), translated from English to the Czech language (Kostalova et al., 2021). The BAASIS consists of five self-report items: one on initiation; three on implementation and one on persistence to the prescribed immunosuppression regimen. The initiation phase item is assessed for co-medications only, as chronic immunosuppression is typically started during the post-transplant inpatient phase. The BAASIS assesses medication adherence for the four weeks preceding the report.

The three implementation items assess “taking” (i.e., missing any dose of medication), “timing” (i.e., taking the medication two hours or more before or after the usual time), and “dosing” (i.e., changing the amount of medication taken without input from a physician). All three either begin with or consist entirely of binary (i.e., yes/no) questions. Any positive answer was considered non-adherence. To evaluate the frequency of implementation problems, positive answers to the “taking” and “timing“ items were followed by five response categories: once, twice, 3 times, 4 times and more than 4 times.

#### 2.4.3 Tacrolimus concentrations

Immunosuppressive regimen details regarding prescribed drugs, dosage forms, dosing schedule and possible switches in drug regimen were abstracted from medical records. Before adherence assessments began, seven tacrolimus trough concentrations were collected over the course of each patient’s scheduled follow-up visits. Based on hospital guidelines, the target range of tacrolimus was 10–15 μg/L in newly transplanted patients and 5–10 μg/L in those at least 30 days post-transplant. Tacrolimus concentrations were calculated based on ethylenediaminetetraacetic acid blood levels measured *via* chemiluminescent microparticle immunoassay in an Architect i1000 analyzer. All tacrolimus trough concentrations included in the IPV calculation were obtained during steady states of tacrolimus therapy, with no dose changes in the 3 days prior to sampling.

Extreme deviations in tacrolimus concentrations were excluded from the analysis if the medical documentation provided explanations (e.g., drug-drug interactions or incorrect administration). If a change was observed from a tacrolimus to a non-tacrolimus-based regimen during the observation period, we noted the reason for the change and included all available tacrolimus values preceding the change.

#### 2.4.4 Intra-patient variability of tacrolimus concentrations

Tacrolimus IPV values were assessed *via* MLVI, CV and time-weighted CV. Both MLVI and CV are calculated from the variance, i.e., s^2^ = Σ(x_i_ – x̄)^2^, where x_i_ is the assay value for observation i, and x̄ is the mean. The MLVI represents the standard deviation of all measured tacrolimus concentrations (i.e., √s^2^). The CV is calculated by multiplying each patient’s MLVI by 100, then dividing the product by the mean tacrolimus concentration, i.e., (100*MLVI) /x̄, thus allowing comparisons between patients with different adherence target levels (Shneider et al., 2018). The calculation of the time-weighted average differed from the non-time-weighted average in that, to determine it, each assay value (x_i_) was multiplied by the time of exposure (t_i_), i.e., half the time interval between the measurement and the value preceding it, plus half the time interval after the measurement. The standard deviation was the square root of the time-weighted variance, i.e., Σ(x_i_–x̄)^2^*t_i_. A detailed explanation of the time-weighted calculations can be found in Rozen-Zvi et al., 2017.

### 2.5 Statistical analysis

Descriptive statistics were applied to summarize variables as appropriate for data type and distribution (e.g., frequencies; percentages; mean/standard deviation; median/interquartile ranges).

The next step was to apply inferential statistics. First, we used generalized additive modeling to explore possible nonlinearities in the association between tacrolimus dosing and its trough concentration. Second, we predicted the dependent variable trough concentration–using random-intercept regression analysis, with additional robust estimation of the standard errors to account for the repeated measurements within patients and, if necessary, the addition of a random slope next to the random intercept. Nonlinearities in the dose-concentration curve were modeled by adding dosing and its higher-order terms as covariates (i.e., tacrolimus dose, its second- (quadratic) and third-order (cubic) parameters), along with the BAASIS score. IPV-derived dependent variables (i.e., logarithmically transformed MLVI, CV and time-weighted CV) were analyzed by ordinary regression analysis.

A *p*-value of <0.05 was considered statistically significant. Analyses were performed in SAS 9.4 (SAS Institute, Cary, NC) and the ‘Mixed GAM Computation Vehicle’ (mgcv) package in R 4.0.0 for the exploration of nonlinearities.

## 3 Results

### 3.1 Socio-demographic and transplant variables

**FIGURE 1:**
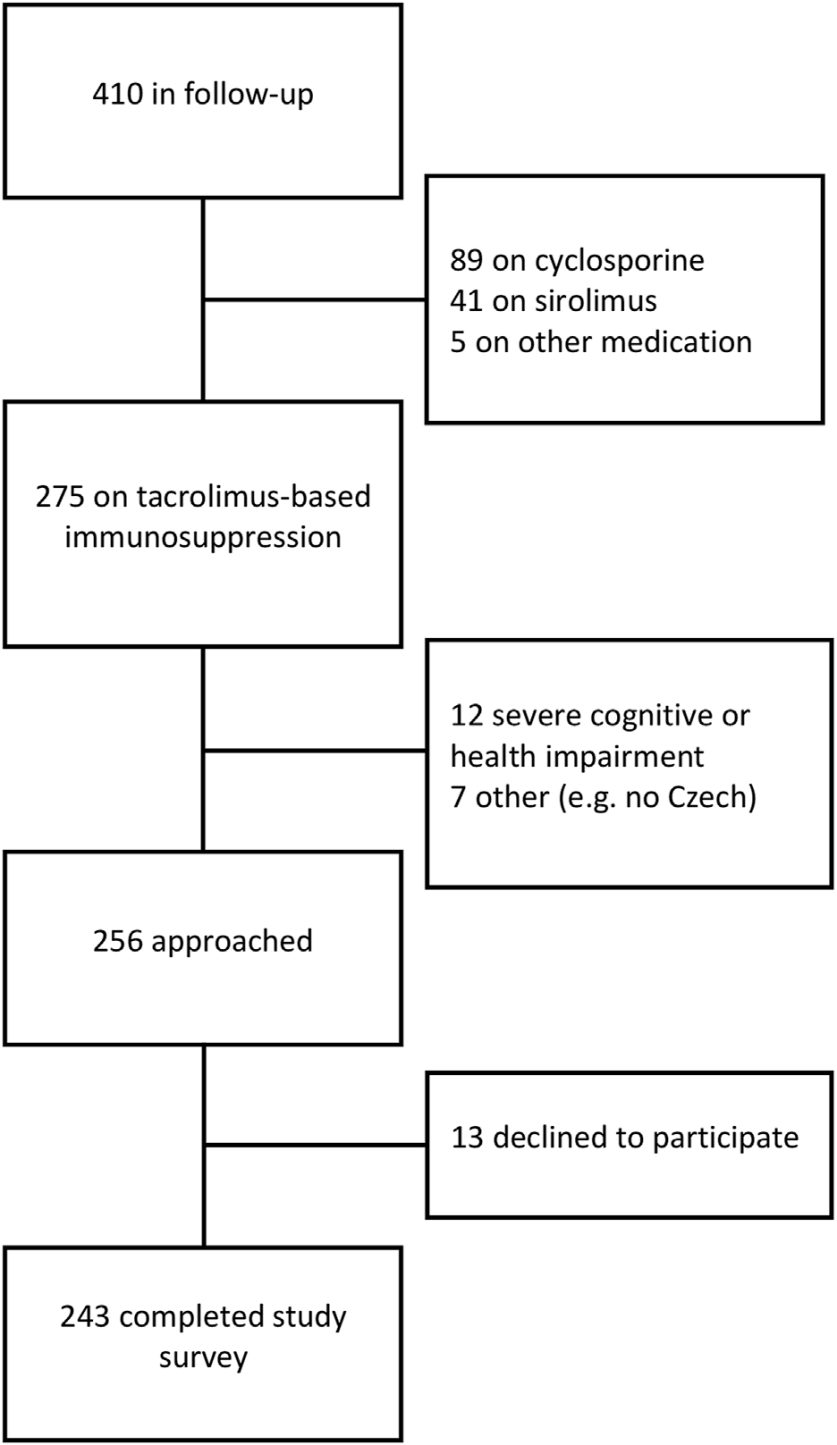
Study population.

Of the 410 patients scheduled to receive regular follow-up care at the post-transplant outpatient clinic until December 2019, 275 were on tacrolimus-based immunosuppression. Based on our inclusion and exclusion criteria (noted above), 256 eligible patients were approached, of whom 243 agreed to participate and completed the survey (Figure 1). Included patients had a median age of 57 years; 165 (67.9%) were male; the median number of years post-transplant was 5.6 (Table 1). Thirty-one (12.8%) were re-transplanted.

### 3.2 Self-reported medication adherence (implementation phase)

Non-adherence to immunosuppressants was found in 102 (42.0%) patients; 35 (14.4%) were non-adherent with the “taking,” 92 (37.9%) with the “timing,” and 1 (0.4%) with the “dosing” aspects of their immunosuppressant regimens.

### 3.3 Tacrolimus concentrations

Most patients (98.8%) took a prolonged-release formulation of tacrolimus; three (1.2%) were treated with immediate-release capsules. Tacrolimus dosage adjustments were made in 102 (42.0%) cases during the observed period: dosages were adjusted once in 79 patients and at least twice in 23 patients.

Seven consecutive tacrolimus concentrations were available for 227 (93.4%) patients. These measurements spanned an average of 14.4 ± 4.5 months (minimum 3 months; maximum 21.5 months). Individual patients’ timespans corresponded with their transplant centers’ care management policies.

Only 6 measurements were available for 15 (6.2%) patients: 10 (4.1%) admitted incorrect administration; 3 (1.2%) were switched from tacrolimus to sirolimus for cancer diagnosis; 1 (0.4%) was switched to a different brand name of tacrolimus extended-release capsule; and 1 (0.4%) discontinued tacrolimus use on physician’s recommendation (because of possible drug-drug interaction). This patient initiated the treatment with diltiazem which is known to be an inhibitor of tacrolimus metabolism. Only 5 measurements were included for 1 (0.4%) patient, who was switched to sirolimus during the observed period.

### 3.4 Intra-patient variability of tacrolimus concentrations

The mean MLVI was 1.54 (median 1.33; SD 0.98; IQR 0.95–1.76); the mean CV was 22.56 (median 20.89; SD 10.82; IQR 15.17–26.51).

Analysis of the association between tacrolimus dose and blood concentrations revealed a nonlinear curve. Significantly lower blood concentrations were found in patients who admitted omission of at least one dose [−0.08; 95% confidence interval (CI) −0.15 to −0.01; *p* = 0.03] or a higher frequency of “timing” problems [−0.02; 95% CI -0.04 to −0.00; *p* = 0.02] (Table 2). However, these associations were not strong: the generalized for both equivalent models using “taking” and “timing” non-adherence as a covariate, our additive modeling approach suggested an *R* ^2^ value of only 3%. Problems with tacrolimus “dosing” could not be evaluated due to their small number of occurrences.

**TABLE 2:** Modeling of blood tacrolimus by the BAASIS^©^, corrected for dosing (*N* = 243)*.

BAASIS question	Estimate	Confidence intervals	Pr > |t|
Taking (yes/no)	−0.0773	−0.1476; −0.0070	0.0313 **
Taking (frequency)	−0.0434	−0.0966; 0.0098	0.1099
Timing (yes/no)	−0.0445	−0.0935; 0.0045	0.0751
Timing (frequency)	−0.0230	−0.0424; -0.0036	0.0201 **

* logarithmically transformed tacrolimus levels to yield a normal distribution, adjusted for tacrolimus dose, dose in quadrate and dose to the third power.

** statistically significant difference from zero (*p*-value <0.05). BAASIS, basel assessment of adherence to immunosuppressive medications scale;N, denominator; Pr > |t|, two-tailed *p*-value computed using the t distribution.

Analysis of IPV variables is shown in Table 3. Significantly higher CVs were found for “taking” non-adherence measured either dichotomously [0.20; 95% CI 0.05–0.36; *p* = 0.01; *R* ^2^ = 3%] or ordinally [0.13; 95% CI 0.01–0.24; *p* = 0.03; *R* ^2^ = 2%]. Also, the time-weighted CVs showed significantly higher variability for dichotomously measured “taking” adherence [0.22; 95% CI 0.02–0.43; *p* = 0.03; *R* ^2^ = 2%].

**TABLE 3:** Modeling of tacrolimus intra-patient variability by the BAASIS (*N* = 243).

	BAASIS^©^ question	Estimate	Confidence intervals	Pr > |t|
MLVI*	Taking (yes/no)	0.1125	−0.0004; 0.2254	0.0507
	Taking (frequency)	0.0749	−0.0077; 0.1575	0.0754
	Timing (yes/no)	−0.0061	−0.0880; 0.0758	0.8828
	Timing (frequency)	−0.0001	−0.0373; 0.0372	0.9973
CV*	Taking (yes/no)	0.2050	0.0501; 0.3599	0.0097 **
	Taking (frequency)	0.1259	0.0120; 0.2399	0.0304 **
	Timing (yes/no)	0.0332	−0.0798; 0.1462	0.5634
	Timing (frequency)	0.0240	−0.02728; 0.0754	0.3271
TWCV*	Taking (yes/no)	0.2245	0.0235; 0.4255	0.0287 **
	Taking (frequency)	0.1340	−0.0136; 0.2816	0.0750
	Timing (yes/no)	0.0200	−0.1251; 0.1681	0.7901
	Timing (frequency)	0.0246	−0.0423; 0.0915	0.4964

* logarithmically transformed to yield a normal distribution.

** statistically significant difference from zero (*p*-value <0.05). BAASIS, basel assessment of adherence to immunosuppressive medications scale; CV, coefficient of variation; MLVI, medication level variability index; N, denominator; Pr >|t|, two-tailed *p*-value computed using the t distribution; TWCV, time-weighted coefficient of variation.

## 4 Discussion

Regular blood level monitoring for calcineurin inhibitors and mTOR inhibitors is available in most transplant centers. However, many transplant centers do not perform standard therapeutic drug monitoring, examining only single drug concentrations at each patient visit (Kidney Disease: Improving Global Outcomes Transplant Work Group, 2009; Shuker et al., 2015). Single concentration testing is valid for a short period after medication intake and may be biased by so called “white coat adherence.” The IPV calculation, which covers multiple drug concentrations, might be more meaningful and less bias-prone surrogate for drug exposure over time. In transplant recipients, higher calculated tacrolimus concentration IPVs have already been associated with negative clinical outcomes including acute rejection, *de novo* donor-specific antibodies formation, graft loss, and mortality (Schumacher et al., 2021).

Comparing various IPV calculations, we found a correlation between self-reported medication non-adherence and higher CVs or time-weighted CVs; however, this relationship did not extend to MLVIs. In a recent systematic review, Schumacher et al. (2021) recommended the CV over other candidates for IPV calculation. The authors’ choice was based on widespread reporting of CV use in the literature, the ease of calculating it, and its standardization for the scale of the dataset. It has also been recommended by various other researchers (Shuker et al., 2015; Kuypers, 2020).

This sample’s median CV was generally comparable to those of other studies: variation ranged from 17.7% (Shuker et al., 2016) to 43.1% (Solomon et al., 2020), but data were mostly concentrated around 23% (Schumacher et al., 2021). For example, the same median of CV (20.5%) was found by Mo et al. in their post-KTx study of 671 patients (Mo et al., 2019). MLVI was also comparable to that calculated by Shemesh et al. in a sample of 379 liver transplant patients [mean 1.7; median 1.3; SD 1.6] (Shemesh et al., 2017).

On the other hand, IPV calculation using time-weighted averages appears to reduce the effect of short periods of multiple measurements (e.g., during hospitalization). Using a study sample of 803 KTx patients, Rozen-Zvi et al. (2017) multivariate analysis showed a clear link between high time-weighted tacrolimus blood concentration CVs and reduced graft survival [hazard ratio 1.74; 95% CI 1.14–2.63; *p* = 0.01]. As a part of our study, we assessed the correlation between time-weighted CV calculation and self-reported medication adherence. Despite their limited use to date, time-weighted CVs also show potential for regular assessment of adherence in clinical practice.

When evaluating IPV calculations’ prognostic value, researchers and clinicians should consider not only inter-measurement intervals but also the time since transplant, as this may also effect the therapeutic value of tacrolimus concentrations (Shuker et al., 2015). Immediately after KTx, factors including the frequent need to adjust dosages (leading to a lack of fixed-target concentrations), or the varying periods patients take to build a stable tacrolimus use routine, IPV calculations appear to have the highest predictive potential when initiated 3–6 months post-transplant. After this period, IPVs better reflect patient medication-taking behavior (Schumacher et al., 2021). For this reason, we included all patients at least 3 months post-transplant.

Another approach assumed a non-linear relationship between tacrolimus trough concentrations and dosing. Using functional regression modeling, a variety of real-world settings (e.g., continuously changing variability over time, irregular observations per patient) could be accommodated. This assumption was tested in a study evaluating data from 960 KTx patients (Kim et al., 2019). In line with that study’s findings, we found a nonlinear function of tacrolimus dose and tacrolimus blood concentrations. Moreover, implementation non-adherence to tacrolimus was associated with lower blood concentrations. Specifically, in line with Kim et al. (2019), we found a direct relationship between the blood tacrolimus level and tacrolimus “taking” and “timing.”

The main limitation of our study was that our instruments lacked the sensitivity to differentiate low-level relationships between IPV calculations and self-reported medication adherence. Regardless of the method used, the explained variability was always around 3%. Weak or even no correlations were also observed in other studies where the IPV of tacrolimus concentration was combined with various methods of adherence measurement such as electronic monitoring (Foster et al., 2018) or self-reports (Foster et al., 2018; Gustavsen et al., 2019; Herblum et al., 2021). Considering the fact that no correlation between IPV and electronic monitoring has yet been found in the literature, IPV should be considered only adherence measure among others. However, the IPV of tacrolimus is probably determined by a set of influencing factors; therefore, it lacks the power to capture medication adherence on its own. This supports Gustavsen et al.‘s recommendation to use multiple tools to capture different patients at risk for non-adherence (Gustavsen et al., 2019).

The retrospective single-centered design also limits our results’ applicability to a broader transplant population. Even though a tacrolimus-based regimen is the therapy of choice, at the time of our study, only 60% of our transplant center’s patients were using tacrolimus (Vankova et al., 2018). Compared with ciclosporin A, tacrolimus is known to have lower individual concentration variability (Heemann and Viklicky, 2017). Therefore, further research should evaluate the IPV calculations when involving patients using numerous types of immunosuppression. We did not include patients on ciclosporin A due to their small number during data collection. Moreover, no more patients are newly initiated with ciclosporin A in our transplant center nowadays.

Our analysis of the association between self-reported adherence and IPV calculation was also limited by the fact that, whereas the BAASIS scale measures adherence to all immunosuppressants for the preceding 4 weeks, tacrolimus blood concentration reflects only a short period after the medication’s intake. There is no gold standard for monitoring adherence in clinical practice. The currently preferred method is combining tools that capture various non-adherent behaviors. Specifically for transplant populations, adherence assessment may be done by combining patient-reported outcome measures with evaluation of immunosuppressant’s trough blood concentration (Gustavsen et al., 2019). We chose the BAASIS scale based on the range of literature using it with transplant populations as well as the fact that its validity has been established in an ongoing validation study (Denhaerynck et al. paper in preparation).

Despite notable limitations, this study showed that high IPVs for tacrolimus concentration reflected implementation non-adherence in KTx recipients. Specifically, the combination of a self-report (e.g., the BAASIS scale) and a CV (calculated using data from medical records), may enable precise regular adherence assessment in clinical practice. Both the BAASIS and the CV are simple, inexpensive, and easy to evaluate. The BAASIS consists of five self-report items, with any positive answer signifying non-adherence. Two versions exist: one is completed as an interview between a healthcare professional and the transplant recipient; the other is a questionnaire that can be completed by transplant recipients on their own. The BAASIS scale is under copyright at the University of Basel. Detailed information about its use can be found on the BAASIS website: https://baasis.nursing.unibas.ch/.

The CV calculation can be incorporated into medical records, making it easy for clinicians to monitor it regularly. High CVs reflect potential non-adherence; however, the definition of “high” varies among studies. Schumacher et al. (2021)’s literature review found that a CV was generally considered high if it was greater than the cohort median or highest quartile. In most studies, CV values of 25% and above were associated with acute rejection at or after 1 year posttransplant.

Due to the high prevalence of non-adherence to immunosuppressants and its negative consequences, it is now recommended to actively screen patients for increased risk for non-adherence (Neuberger et al., 2017). In any case where potential medication non-adherence is identified by a high tacrolimus concentration IPV, the patient should be questioned about possible influencing factors such as drug administration in relation to food, recent acute diseases (e.g., diarrhea), and the use of possible interacting agents (e.g., initiation of new medication by another physician, self-medication and known interactive nutrients).

Patients at risk for non-adherence should be targeted with adherence-enhancing interventions and their adherence redetermined in association with calculated IPV and self-reports (Herblum et al., 2021). To date, no randomized controlled trial has been found on this topic (Schumacher et al., 2021).

## 5 Conclusion

Immunosuppressant implementation non-adherence was reflected by higher CVs or time-weighted CVs of tacrolimus concentration, as well as lower concentrations in the blood. Simple IPV calculations incorporated into medical records should be considered for everyday clinical practice as an additional tool to identify patients at risk for non-adherence.

## Data Availability

The raw data supporting the conclusions of this article will be made available by the authors, without undue reservation.

## Footnotes

1 Data of the Coordination Center for Transplantation. https://www.kst.cz/en/[Accessed 25 May 2021]

## Abbreviations

95% CI, 95% confidence interval; CV, coefficient of variation; IPV, intra-patient variability; KTx, kidney transplantation; MLVI, medication level variability index.
